# The complete mitochondrial genome of the forest crested lizard, *Calotes emma* (Squamata, Agamidae) in China by the next generation sequencing

**DOI:** 10.1080/23802359.2021.2008822

**Published:** 2021-12-19

**Authors:** Wenfang Ma, Xiaotong Jing, Xiaomei Wei, Yong Huang

**Affiliations:** a Guangxi University of Chinese Medicine, Nanning, Guangxi, China; b Guangxi Botanical Garden of Medicinal Plants, Nanning, Guangxi, China

**Keywords:** Agamidae, *Calotes emma*, mitochondrial genome, next-generation sequencing, phylogenetic tree

## Abstract

The whole mitogenome can prove useful tools for phylogenetic reconstruction and efficiently recover with reasonable taxon sampling. *Calotes emma* is widely distributed and arboreal in habits. However, studies of *C. emma* are still very limited, including population genetics and evolutionary biology. In this study, we reported the complete mitochondrial genome of the *C. emma* by next-generation sequencing for future more researches on systematics and evolution of *C. emma* from the perspective of mitochondrial DNA. The length of mitogenome was 17,688 bp, including 13 protein-coding genes (PCGs), 2 ribosomal RNA (rRNA) genes, 22 tRNA genes and a control region. The phylogenetic tree recovered the monophyly of the *Calotes* and revealed that newly sequenced *C. emma* well supported as the sister taxon to *C. mystaceus* by very high posterior probabilities (1.0). The complete mitochondrial genome of *C.emma* in this study will be helpful for understanding the phylogenetic systematics and relationships, and molecular evolution of *Calotes* in Agamidae.

Lizards of the genus *Calotes* Cuvier, 1817, belonging to the family Agamidae in the order Squamata, currently include 26 species distributed from eastern Iran through south China to Sumatra, Indonesia (Vindum et al. 2003; Zug et al. 2006; Krishnan 2008; Hartmann et al. 2013; Amarasinghe, Karunarathna and Fujinuma 2014; Amarasinghe, Karunarathna, Hallermann et al. 2014). The forest crested lizard, *Calotes emma* Gray,1845, one of the typical members of the genus *Calotes*, is widely distributed across from southern China, India, Vietnam, Burma, Lao PDR, Thailand, Cambodia to Peninsular Malaysia (Zhao et al. 1999; Chan-Ard et al. 2015). This species is diurnal and arboreal in habits, and prefers in tropical forests or open areas under forests at elevations of 80–1950 m (Zhao et al. 1999; Agarwala and Majumder 2015). *C. emma* blend in perfectly with their surroundings, but it is usually found along stream margins of secondary mixed moist deciduous forests and in semi-evergreen forests (Agarwala and Majumder 2015). Grasshoppers, ants, termites, cockroaches, beetles, diverse species of moths and low flying butterflies, and soil-living insects and their larvae are food for *C. emma* (Agarwala and Majumder 2015).

Study of *C. emma* are still very limited, including population genetics and evolutionary biology. To date, there is only one study that high genetic differences among populations of *C. emma* was found throughout Thailand using the mitochondrial CO1 (Saijuntha et al. 2020). Mitochondrial DNA, as an ideal molecular marker, has been widely used in the study of population genetics and evolution. In addition, the whole mitogenome can prove more credible results for phylogenetic reconstruction and efficiently recover with reasonable taxon sampling than single gene (Rubinstein et al. 2013; Yuan et al. 2016). However, the complete mitogenome sequences of *C. emma* is little known. Thus, in order to provide more data for systematics and evolution of *C. emma* for future researches from the perspective of mitochondrial DNA, in this study, we reported the complete mitogenome of *C. emma* using next-generation sequencing, and then compared with the other agamid lizards which their mitogenomes have been sequenced. It will provide insight into exploring the phylogenetic relationships and evolution of *Calotes* in Agamidae.

The specimen of *C. emma* was sampled from the locality of Pingbian (N22.9617, E103.7917), Yunnan Province, China. Its muscle issue is fixed with 95% ethanol and stored at −20 °C in the herpetological collection, Guangxi University of Chinese Medicine(http://www.gxtcmu.edu.cn/, Yong Huang, huangykiz@163.com) under voucher number 201607241. Total genomic DNA was extracted from the muscle for subsequent analyses using the TIANamp Genomic DNA kit (Tiangen Biotech (Beijing) CO., LTD) and then the complete mitogenome was sequenced by an Illumina Hiseq 2000 platform (Illumina, San Diego, CA, USA). We firstly used fastqc (http://www.bioinformatics.babraham.ac.uk/projects/fastqc/) to evaluate the sequencing results, and then controlled the sequencing quality by ngsqc software. We then performed SPAdes v3.11.0 (Bankevich et al. 2012) to d*e novo* to assemble the 18,425,455 clean reads. Finally, we obtained the complete mitogenome sequence of *C. emma* using the mitogenome of *C.mystaceus* (GenBank accession number MT872513; Wang et al. 2020) and *C. versicolor* (GenBank accession number NC_009683; Amer and Kumazawa 2007) as references. The annotation and direction of mitogenome genes were inferred by the MITOS web server (Bernt et al. 2013).

The circular mitogenome of *C.emma* (MZ359215) is 17,118 bp in length, including 13 protein-coding genes (PCGs), 2 ribosomal RNA (rRNA) genes, 22 tRNA genes and a control region. Seven tRNA (tRNA- Gln, Ala, Asn, Cys, Ser, Tyr, and Glu) and Nad6 were encoded on the L-light strand, and the others (12 PCGs and 15 tRNA) were encoded on the H-heavy strand. The overall base composition of the mitogenome is 33.36%A, 27.74%G, 13.52%C and 25.38%T. Eleven PCGs were initiated with the ATG codon, but ND4L begins with ATA and ND5 begins with ATC. Meanwhile, there PCGs (ND1, ND2 and ND6) were ended with TAG codon, five PCGs (COX1, COX2, ATP8, ND4L and Cytb) were terminated with TAA codon, ND4 stopped with AGG, ND5 stopped with AGA, and three PCGs (COX3, ND3 and ATP6) were ended with an incomplete termination base T. The length of D-loop, 12S rRNA and 16S rRNA were 1,444 bp, 829 bp, and 1,524 bp, respectively. The length of 22 tRNA genes ranged from 56 bp in tRNA-Cys to 75 bp in tRNA-Leu.

We constructed phylogenetic analysis using 25 mitogenomes of the Anguidae, Crocodylidae, Anguinae, Gerrhonotinae and Helodermatidae lizards using *Shinisaurus crocodilurus* (Shinisauridae) as outgroup taxa. We performed the Bayesian inference with GTR + G + I model of nucleotide substitution by MrBayes v.3.2.2 (Ronquist et al. 2012). The most appropriate evolutionary nucleotide substitution model of the 13 PCGs was selected by PartitionFinder 2. The phylogenetic tree (Figure 1) recovered the monophyly of *Calotes* and revealed that newly sequenced *C.emma* well supported as the sister taxon to *C. mystaceus* by very high posterior probabilities (1.0). The complete mitochondrial genome of *C.emma* will be helpful for understanding phylogenetic systematics and relationships, and molecular evolution of *Calotes* in Agamidae.

**Figure 1.**
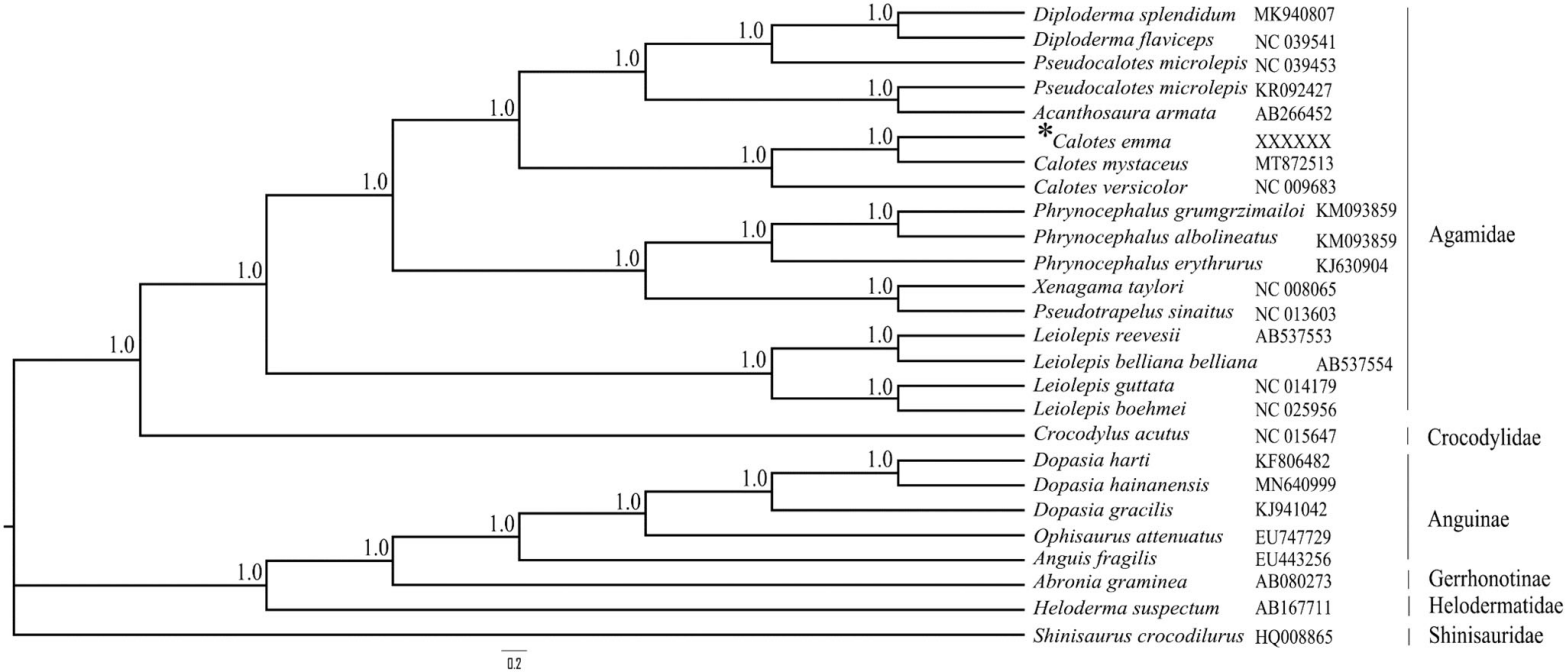
A phylogenetic tree inferred from Bayesian inference of 25 lizards based on 13 protein-coding genes. Node numbers show Bayesian posterior probabilities. The GenBank accession numbers are shown following the names of species.

## Data Availability

The genome sequence data that support the findings of this study are openly available in GenBank of NCBI (https://www.ncbi.nlm.nih.gov/) under the accession no. MZ359215. The associated BioProject, SRA, and Bio-Sample numbers are PRJNA773849, SRR16596561, and SAMN22514025 respectively.

## References

[CIT0001] Agarwala BK, Majumder J. 2015. *Calotes emma* Gray, 1845 (Squamata: Agamidae): range extension and new addition to the reptilian fauna of Tripura, northeast India. Check List. 11(2):1562.

[CIT0002] Amarasinghe AAT, Karunarathna DMSS, Fujinuma J. 2014. A new *Calot*es species from Sri Lanka with a redescription of *Calotes liolepis* Boulenger, 1885. Herpetologica. 70 (3):323–338.

[CIT0003] Amarasinghe AAT, Karunarathna DMSS, Hallermann J, Fujinuma J, Grillitsch H, Campbell PD. 2014. A new species of the genus *Calotes* (Squamata: Agamidae) from high elevations of the Knuckles Massif of Sri Lanka. Zootaxa. 3785 (1):59–78. 24872171 10.11646/zootaxa.3785.1.5

[CIT0004] Amer SA, Kumazawa Y. 2007. The mitochondrial genome of the lizard *Calotes versicolor* and a novel gene inversion in South Asian draconine agamids. Mol. Biol. E. 24 (6):1330–1339. 10.1093/molbev/msm054 17379622

[CIT0005] Bankevich A, Nurk S, Antipov D, Gurevich A, Dvorkin M, Kulikov AS, Lesin V, Nikolenko S, Pham S, Prjibelski A, et al. 2012. SPAdes: a new genome assembly algorithm and its applications to single-cell sequencing. J Comput Biol. 19(5):455–477. 22506599 10.1089/cmb.2012.0021 PMC3342519

[CIT0006] Bernt M, Donath A, Jühling F, Externbrink F, Florentz C, Fritzsch G, P€Utz J, Middendorf M, Stadler PF. 2013. MITOS: improved de novo metazoan mitochondrial genome annotation. Mol Phylogenet Evol. 69(2):313–319. 22982435 10.1016/j.ympev.2012.08.023

[CIT0007] Chan-Ard T, Parr JWK, Nabhitabhata J. 2015. A field guide to the reptiles of Thailand. Oxford University Press, New York.

[CIT0008] Hartmann T, Geissler P, Poyarkov NA, Ihlow F, Galoyan EA, Rödder D, Böhme W. 2013. A new species of the genus *Calotes* Cuvier, 1817 (Squamata: Agamidae) from southern Vietnam. Zootaxa. 3599:246–260. 24613873 10.11646/zootaxa.3599.3.3

[CIT0009] Krishnan S. 2008. New species of *Calotes* (Reptilia: Squamata: Agamidae) form the Southern Western Ghats, India. J Herpetol. 42(3):530–535.

[CIT0010] Ronquist F, Teslenko M, van der Mark P, Ayres DL, Darling A, H€Ohna S, Larget B, Liu L, Suchard MA, Huelsenbeck JP. 2012. MrBayes 3.2: efficient Bayesian phylogenetic inference and model choice across a large model space. Syst Biol. 61(3):539–542. 22357727 10.1093/sysbio/sys029 PMC3329765

[CIT0011] Rubinstein ND, Feldstein T, Shenkar N, Botero-Castro F, Griggio F, Mastrototaro F, Delsuc F, Douzery EJP, Gissi C, Huchon D. 2013. Deep sequencing of mixed total DNA without barcodes allows efficient assembly of highly plastic ascidian mitochondrial genomes. Genome Biol Evol. 5(6):1185–1199. 23709623 10.1093/gbe/evt081 PMC3698926

[CIT0012] Saijuntha W, Tantrawatpan C, Pilap W, Sedlak S, Sakdakham K, Sripirom P, Suksavate W, Kongbuntad W,T awong W. 2020. Genetic differentiation of the forest crested lizards, *Calotes emma alticristatus* Schmidt, 1925 and *C. emma emma* Gray, 1845 (Squamata: Agamidae) relating to climate zones in Thailand.Asian Herpetological Research,11(1):19–27.

[CIT0013] Vindum JV, Win H, Thin T, Lwin KS, Shein AK, Tun H. 2003. A new *Calotes* (Squamata: Agmidae) from the Indo-Burman range of western Myanmar (Burma). Proc Calif Acad Sci. 54(1):1–16.

[CIT0014] Wang M, Jiang Z, Wang J, Cui L, Zhang M. 2020. The complete mitochondrial genome of the blue-crested lizard, *Calotes mystaceus* (Squamata, Agamidae) in China. Mitochondrial DNA B Resour. 5 (3):3530–3531. 10.1080/23802359.2020.1822219 PMC7782218 33458223

[CIT0015] Yuan S, Yun X, Zheng Y, Zeng XM. 2016. Next-generation sequencing of mixed genomic DNA allows efficient assembly of rearranged mitochondrial genomes in Amolops chunganensis and Quasipaa boulengeri. Peerj. 4(3):e2786. 27994980 10.7717/peerj.2786 PMC5162401

[CIT0016] Zhao EM, Zhao KT, Zhou KY, et al. 1999. Fauna Sinica, Rep-tilia, Vol. 2: Squamata, Lacertilia. Beijing: Science Press.

[CIT0017] Zug GR, Brown HHK, Schulte JA, II, Vindum JV. 2006. Systematics of the garden lizards, *Calotes versicolor* Group (Reptilia, Squamata, Agamidae), in Myanmar: central dry zone populations. Proc Calif Acad Sci. 57(2):35–68.

